# Supplementary material for: Content-rich biological network constructed by mining PubMed abstracts
Source: BMC Bioinformatics. 2004 Oct 8;5:147. doi: 10.1186/1471-2105-5-147 (PMC528731; doi:10.1186/1471-2105-5-147)
Supplement: Additional File 2 — The original results of the above study (non-essential files are deleted to keep the file size under the limit set by BMC bioinformatics). [file 1471-2105-5-147-S2.bz2 › chilibotAdditionalFile2/dip50/648ID7922031E11035/html/CGA_CGB.html]

 


 **CGA** and **CGB** 
  
Found 505 abstracts in PubMed, retrieved 50.  
 

 What does Google say? 
 PDF only 
| .edu only 

---

**Interactive relationship** (e.g. stimulation, inhibition, etc)

**Neutral relationship**- Cross reactions of anti human chorionic gonadotropin beta  [ **CGB** ]  antibody elicited by HCG  [ **CGA** ]  beta based antifertility vaccines with other autoantigens may occur due to identical or homologous protein subsequences.  Ref: 12687122 Russ J Immunol< MedlineTA>Russ J Immunol,
- The expression of  **CgA**  and  **CgB**  was higher in NIDCs than in IDCs  **CgA**  = 70% of NIDC vs 22% of IDC and  **CgB**  = 65% of NIDC vs 30% of IDC.  Ref: 12165659 Endocr Pathol< MedlineTA>Endocr Pathol,
- Nested reverse transcriptase polymerase chain reaction using primers specific for beta human chorionic gonadotropin beta  [ **CGB** ]  HCG  [ **CGA** ]  or alpha fetoprotein AFP detected 1 beta HCG  [ **CGA** ]  or AFP producing cell in 1 x 10 6 PBSCs.  Ref: 11832775 J Urol, 2002
- In addition, transfection of  **CGA**  and  **CGB**  into nonneuroendocrine COS 7 and NIH3T3 cells repeatedly indicated the nuclear localization of  **CGB**  in addition to its usual localization in the cytoplasm.  Ref: 11854265 J Biol Chem, 2002
- In adult islets, alpha cell granules stored  **CgA**  in the halo and  **CgB**  in both the core and the halo, beta cell granules stored both  **CgA**  and  **CgB**  in their cores, and in delta cell granules, both substances were mixed throughout the matrix.  Ref: 12826904 Pancreas, 2003
- Hybrid hormone preparations were prepared by combining intact and Asn 56 deglycosylated N 56 dg equine e LH or FSH alpha  [ **CGA** ]  subunit preparations with truncated, des 121 149 eLH beta eLH beta t, immunopurified, intact eLH beta or equine chorionic gonadotropin beta  [ **CGB** ]  eCG beta preparations, and eFSH beta.  Ref: 11874703 J Endocrinol, 2002

**Non-interactive relationship** (e.g. studied together, co-existance, homology, etc.)

- Chromogranin A  **CgA**  and chromogranin B  **CgB**  were positive in 21 26% and 30 cases 38% respectively.  Ref: 12375049 Oncol Rep< MedlineTA>Oncol Rep, 2002
- While alpha gonadotrophin subunit alpha GSU, SgII and  **CgA**  mRNA levels did not change,  **CgB**  mRNA levels were elevated in EF ewes and had declined in ewes around the surge.  Ref: 12176657 J Endocrinol, 2002
- This was done to differentiate the cells into a phenotype more like syncytiotrophoblast cells as evidenced by production of beta human chorionic gonadotropin beta  [ **CGB** ]  hCG  [ **CGA** ] .  Ref: 12517328 Hypertens Pregnancy, 2002
- The unusual chromogranin B  **CgB**  chromogranin A  **CgA**  ratio with  **CgB**  in abundance over  **CgA** , previously found only in SP, was also valid for the native NP and metastasis derived prostasomes MP.  Ref: 12539232 Prostate, 2003
- Insulin was expressed in early fetal cells of both pigs and humans, and colocalization with  **CgA** ,  **CgB** , and synaptophysin was demonstrated.CONCLUSION The early expression of  **CgA**  and synaptophysin may reflect a role at early fetal stages, and the individual localization of  **CgA**  and  **CgB**  upon differentiation indicates individual functions.  Ref: 12826904 Pancreas, 2003
- Serum beta human chorionic gonadotropin beta  [ **CGB** ]  HCG  [ **CGA** ]  was serially increased 19.71 22.71 mIU mL.  Ref: 12186103 Am J Med Sci, 2002
- CASE REPORT A 36 year old woman with stage IB 1 squamous cell carcinoma of the uterine cervix and a high serum beta human chorionic gonadotropin beta  [ **CGB** ]  hCG  [ **CGA** ]  level underwent radical hysterectomy with pelvic and para aortic lymphadenectomy.  Ref: 12477457 Gynecol Oncol, 2002
- With the association of some biochemical markers like maternal serum free beta human chorionic gonadotropin beta  [ **CGB** ]  hCG  [ **CGA** ]  and pregnancy associated plasma protein A PAPP A to ultrasonography at 11 14 weeks, it is possible to identify about 90% of chromosomal abnormalities.  Ref: 12635737 Clin Exp Obstet Gynecol, 2002
- With multivariate analysis, expression of BRCA1 significantly influenced the expression of  **CgA**  p=0.01, while no factor significantly influenced to the expression of  **CgB** .  Ref: 12375049 Oncol Rep< MedlineTA>Oncol Rep, 2002
- Recent advances in chromogranin research include establishing the role of  **CgA**  in granulogenesis and the role of  **CgB**  in nuclear transcription.  Ref: 12746559 Endocr Pathol, 2003
- new biologic activities for  **CgA** ,  **CgB** , and SgII derived peptides.  Ref: 12746559 Endocr Pathol, 2003
- In the presence of placental remnants, blood was sampled for measurement of beta human chorionic gonadotropin beta  [ **CGB** ]  hCG  [ **CGA** ] , hemoglobin Hb and infectious parameters.  Ref: 12493048 Ultrasound Obstet Gynecol, 2002
- Associations between granins secretogranin II SgII and chromogranin A and B  **CgA**  and  **CgB**  and gonadotrophins LH and FSH have been reported in rodents and they may interact to facilitate differential storage and secretion of LH and FSH.  Ref: 12176657 J Endocrinol, 2002
- In addition to the NT, maternal serum markers as the free beta human chorionic gonadotropin beta  [ **CGB** ]  hCG  [ **CGA** ]  and pregnancy associated plasma protein PAPP A protein can improve the detection rate to 80% with a fixed 5% of FPR.  Ref: 12661334 Ginecol Obstet Mex, 2002
- Unlike  **CgA** , another granin protein  **CgB**  could not substitute for the role of  **CgA**  in secretory granule biogenesis.  Ref: 12438143 Ann N Y Acad Sci, 2002
- To learn the possible roles of chromogranin in breast carcinoma, we immunohistochemically investigated tissue localization of chromogranin A  **CgA**  and chromogranin B  **CgB**  in 10 normal breast tissues, 23 noninvasive ductal carcinomas NIDCs, and 169 invasive ductal carcinomas IDCs and compared their expression with estrogen receptor ER, progesterone receptor PR, and Ki67.  Ref: 12165659 Endocr Pathol< MedlineTA>Endocr Pathol, 2002
- OBJECTIVE Pure germinomas have a good prognosis compared to those with syncytiotrophoblastic giant cells STGCs, which show elevated human chorionic gonadotropin or human chorionic gonadotropin beta  [ **CGB** ]  hCG  [ **CGA** ]  hCG  [ **CGA** ]  beta levels and relapse frequently.  Ref: 12434171 Acta Neurochir Wien , 2002
- We found that  **CgB** , but not  **CgA** , immunoreactivity was selectively associated with prion protein deposits.  Ref: 12692477 Neuroreport, 2003
- Here the effects of several insulin and insulin like growth factor I IGF I doses, either singly or in synergy with serum, on human chorionic gonadotropin beta  [ **CGB** ]  hCG  [ **CGA** ]  beta secretion RIA, proliferation cell counting, cyclin B 1 levels and invasion Matrigel invasion assay, secretion of matrix metalloproteinases MMP 2 and 9 were investigated.  Ref: 12029486 Histochem Cell Biol, 2002
- This study was designed to evaluate the monitoring impact of urinary beta human chorionic gonadotropin beta  [ **CGB** ]  HCG  [ **CGA** ]  on tumor growth in orthotopic implantation model.  Ref: 12508539 Ai Zheng, 2002
- For a 5% false positive rate, the sensitivities are approximately 30% for maternal age alone, 60 70% for maternal age and second trimester maternal serum biochemical testing, 75% for maternal age and first trimester fetal nuchal translucency NT scanning, and 90% for maternal age with fetal NT and maternal serum free beta human chorionic gonadotropin beta  [ **CGB** ]  hCG  [ **CGA** ]  and pregnancy associated plasma protein A PAPP A at 11 14 weeks.  Ref: 12422904 J Matern Fetal Neonatal Med, 2002
- The first step in the diagnosis of ectopic pregnancy is demonstration of pregnancy by means of a rapidly performed and sensitive qualitative urine test for the beta subunit of human chorionic gonadotropin beta  [ **CGB** ]  hCG  [ **CGA** ] .  Ref: 12085079 Ann Emerg Med, 2002
- On the other hand, there was a significant correlation between expression of  **CgB**  and PR and an inverse correlation between  **CgA**  and Ki67 in IDCs of overall cases.  Ref: 12165659 Endocr Pathol< MedlineTA>Endocr Pathol, 2002
- OBJECTIVE The purpose of this study was to evaluate whether abnormal levels of first trimester maternal serum free human chorionic gonadotropin beta  [ **CGB** ]  hCG  [ **CGA** ]  are predictive of adverse pregnancy outcomes.  Ref: 12393965 Fetal Diagn Ther< MedlineTA>Fetal Diagn Ther, 2002
- **CgA**  and  **CgB**  may mediate tumor progression through some unknown function besides the BRCA1 related function.  Ref: 12375049 Oncol Rep< MedlineTA>Oncol Rep, 2002
- ISSUES Two first trimester maternal serum screening markers, pregnancy associated plasma protein A PAPP A and free beta human chorionic gonadotropin beta  [ **CGB** ]  hCG  [ **CGA** ] , are useful for identifying women at increased risk for fetal Down syndrome.  Ref: 12204419 Clin Chim Acta, 2002
- Strategies for diagnosing ectopic pregnancy that defer endovaginal ultrasound in women with suggestive symptoms and serum beta human chorionic gonadotropin beta  [ **CGB** ]  hCG  [ **CGA** ]  levels less than 00 mIU mL ignore the increased risk of ectopic pregnancy in these patients.  Ref: 12574008 Acad Emerg Med, 2003
- Recently it was found that the secretory granules contain three isoforms of inositol, 5 trisphosphate receptor IP 3 R Ca 2 channels and high capacity, low affinity Ca 2 storage proteins chromogranins A  **CgA**  and B  **CgB** .  Ref: 12438140 Ann N Y Acad Sci, 2002
- RESULTS The 14 patients with a pregnancy on the cicatrix of a previous cesarean section at the uterine isthmus in the first trimester were painless, had slight vaginal bleeding, and concurrently had increased serum beta subunit human chorionic gonadotropin beta  [ **CGB** ]  HCG  [ **CGA** ] .  Ref: 11940341 Chin Med J Engl , 2002
- Expression of both  **CgA**  and  **CgB**  was not correlated with age, tumor size, and treatment modality.  Ref: 12375049 Oncol Rep< MedlineTA>Oncol Rep, 2002
- The chromogranin family includes chromogranins A  **CgA**  and B  **CgB**  and secretogranin II SgII, once called chromogranin C.  Ref: 12746559 Endocr Pathol, 2003
- INTRODUCTION AND AIMS To get more insight into the differentiation patterns of pancreatic islet neuroendocrine cells and granules during ontogeny, the expression and localization of chromogranin A  **CgA** , chromogranin B  **CgB** , synaptophysin, and insulin were ultrastructurally studied with the immunogold technique in porcine and human pancreatic islet neuroendocrine cells.METHODOLOGY AND RESULTS In porcine pancreas at early fetal stage,  **CgA**  was visualized throughout the immature granules in all neuroendocrine cells.  Ref: 12826904 Pancreas, 2003
- Chromogranins A  **CGA**  and B  **CGB**  are two major Ca 2 storage proteins of the secretory granules of neuroendocrine cells.  Ref: 11854265 J Biol Chem, 2002
- **CGA**  and  **CGB** , more commonly viewed as markers for endocrine and neuronal cells, were also found to have an immune origin.  Ref: 12091348 Blood, 2002
- These molecules derived from  **CGA**  and  **CGB** , respectively, possess antimicrobial properties.  Ref: 12091348 Blood, 2002
- Chromogranin A  **CGA**  and chromogranin B  **CGB**  are acidic proteins stored in secretory organelles of endocrine cells and neurons.  Ref: 12091348 Blood, 2002
- The immunoreactive patterns of  **CgA**  and  **CgB**  were investigated in CJD and compared to those observed in Alzheimer s disease.  Ref: 12692477 Neuroreport, 2003
- A blood sample for hemoglobin and serum beta human chorionic gonadotropin beta  [ **CGB** ]  hCG  [ **CGA** ]  was obtained on day 1 and the hemoglobin level checked again on day 7.  Ref: 12039111 Obstet Gynecol, 2002
- The data suggest that  **CgA**  and  **CgB**  may play some role in the early phase of neoplastic progression.  Ref: 12165659 Endocr Pathol< MedlineTA>Endocr Pathol, 2002
- We report on a 30 year old female patient with a beta human chorionic gonadotropin beta  [ **CGB** ]  HCG  [ **CGA** ]  producing lung tumour.  Ref: 11863101 Ann Oncol, 2002
- **CgA**  and  **CgB**  were sporadically detected in normal cells of the ducts, acini, and luminal secretion.  Ref: 12165659 Endocr Pathol< MedlineTA>Endocr Pathol, 2002
- An osteolytic meningioma in a 36 year old woman was accompanied by elevated serum levels of human chorionic gonadotropin beta  [ **CGB** ]  subunit beta HCG  [ **CGA** ] , which returned to normal after removal of the tumor.  Ref: 12134912 J Neurosurg, 2002
- **CGA**  overexpression was not accompanied by overexpression of the  **CGB** , LHB, TSHB or FSHB genes to produce ectopic glycoprotein hormones.  Ref: 12180238 Eur Urol, 2002
- RESULTS The 15 patients had light, painless vaginal bleeding and their serum beta subunit human chorionic gonadotropin beta  [ **CGB** ]  HCG  [ **CGA** ]  was elevated.  Ref: 12065130 Int J Gynaecol Obstet, 2002
- The IP 3 R Ca 2 channel was shown to directly interact with  **CgA**  and  **CgB**  at the intragranular pH 5.5, and this coupling led to modulation of the IP 3 R Ca 2 channel activity by the coupled chromogranins.  Ref: 12438140 Ann N Y Acad Sci, 2002
- The patient declined surveillance with serial beta human chorionic gonadotropin beta  [ **CGB** ]  hCG  [ **CGA** ]  levels and was lost to follow up.  Ref: 12738129 Obstet Gynecol, 2003
- In contrast to  **CGB**  1 40,  **CGA**  1 40 and  **CGA**  1 76 VS I both displayed significant intrinsic vasodilator effects.  Ref: 11891009 Regul Pept, 2002
- N terminal peptides of chromogranin A and B  **CGA**  and  **CGB**  were compared for dilator responses in isolated bovine coronary arteries bCoA, measuring diameter changes as a function of pressure.  Ref: 11891009 Regul Pept, 2002
- Primary tumors were analyzed for p53 expression using IHC staining and for beta human chorionic gonadotropin beta  [ **CGB** ]  hCG  [ **CGA** ] , hepatocyte growth factor receptor c Met, and universal melanoma antigen uMAGE messenger RNA expression using reverse transcriptase polymerase chain reaction and electrochemiluminescence.  Ref: 12470104 Arch Surg, 2002
- RNA messengers coding for  **CGB**  were amplified by reverse transcription polymerase chain reaction in human monocytes, and immunocytochemical analysis by confocal microscopy revealed the presence of  **CGA**  or  **CGB**  or both in monocytes and neutrophils.  Ref: 12091348 Blood, 2002
- Diagnosis is still limited by following persistently elevated or rising postevacutation beta human chorionic gonadotropin beta  [ **CGB** ]  hCG  [ **CGA** ]  titers.  Ref: 12658908 Wien Klin Wochenschr, 2003
- Beta human chorionic gonadotropin beta  [ **CGB** ]  HCG  [ **CGA** ]  secreting germ cell tumors are one of the sources that have to be considered in the differential diagnosis of processes inducing a peripheral precocious puberty.  Ref: 12123344 Ann Oncol, 2002
